# Habitat Characteristics and Eggshell Distribution of the Salt Marsh Mosquito, *Aedes vigilax*, in Marshes in Subtropical Eastern Australia

**DOI:** 10.1673/031.008.2501

**Published:** 2008-03-25

**Authors:** Pat E. R. Dale, Jon Knight, Brian H. Kay, Heather Chapman, Scott A. Ritchie, Michael D. Brown

**Affiliations:** ^1^ Griffith School of Environment, Griffith University, Nathan, Brisbane, Australia 4111; ^2^ Queensland Institute of Medical Research, PO Box Royal Brisbane Hospital, Herston, Brisbane, Australia 4029; 3 Tropical Public Health Unit, Queensland Health, PO Box 1103, Cairns, Australia 4870; 4 Current address: National Research Centre for Environmental Toxicology (EnTox), University of Queensland, Australia

**Keywords:** oviposition, intertidal wetlands, environmental characteristics

## Abstract

Research at 10 locations in coastal subtropical Queensland, Australia, has shown that salt marshes contained heterogeneous distributions of eggshells of the pest and vector mosquito *Aedes vigilax* (Skuse) (Diptera:Culicidae). The eggshell distribution was related to specific vegetation assemblages, with a mix of the grass, *Sporobolus virginicus* (L.) Kunth (Poales: Poaceae), and the beaded glasswort, *Sarcocornia quinqueflora* (Bunge ex (Ung.-Stern) A.J. Scott (Caryophyllales: Chenopodiaceae), as significantly higher in eggshells than any other vegetation. There were also high numbers in the mix of *S. virginicus* with the arrowgrass, *Triglochin striata* Ruiz & Pavón (Alismatales: Juncaginaceae). Both mixed types are found in relatively wetter areas, despite very few eggshells being found generally in the low marsh. Most sites contained *S. virginicus* and eggshell locations were variable for this species alone. This was probably related to its life form variability in response to salinity and location on the marsh. Location on the marsh was important for eggshell distribution with most eggshells around the edges of pools and depressions, followed by, but to a significantly lesser extent, the marsh surface. Eggshells were fewest in the low marsh. Partition analysis resulted in a tree that simplified and summarised the factors important for eggshell distribution confirming the individual analyses. The potential effects of climate, sea level and other change are also briefly discussed in the context of likely changes to land cover and relative location on the marsh. For example, increased sea level may lead to low marsh conditions extending into higher marsh area with implications for oviposition and numbers of eggshells.

## Introduction

*Aedes vigilax* (Skuse) (Diptera: Culicidae) is a salt marsh mosquito common in intertidal wetlands in Australia. It is of medical significance as it transmits Ross River virus, a polyarthritis disease with between 4000 and 8000 reported cases each year in Australia. The majority of reported cases are in Queensland, a subtropical to tropical state (Russell 2002). Mosquito management is a major part of controlling the disease. A preliminary requirement for management is to identify the habitats of the immature mosquito since control is most efficiently carried out at the larval stage when the spatial distribution is limited to water bodies. While larval survey is common it can only be done when pools are holding water. At other times assessing the distribution of *Aedes vigilax* eggshells is a good indicator of oviposition activity, as it appears to be relatively stable both spatially and temporally (Ritchie 1994). This means that the timing of surveys is less important. If general patterns are established then these can be used to focus field survey and control on habitats likely to contain larvae during flooding. Several methods of extracting eggshells from substrate samples have been described (Service 1993). One of the simplest ways is by sieving and flotation. The extraction method involves subsampling (Ritchie 1994; Ritchie and Jennings 1994). Although this does not recover all the eggshells compared to direct examination, little information appears to be lost (Turner and Streever 1997a). Indeed simply recording the presence of eggshells may be a sufficient indication of breeding for operational purposes (Dale et al. 1999).

**Figure 1.**
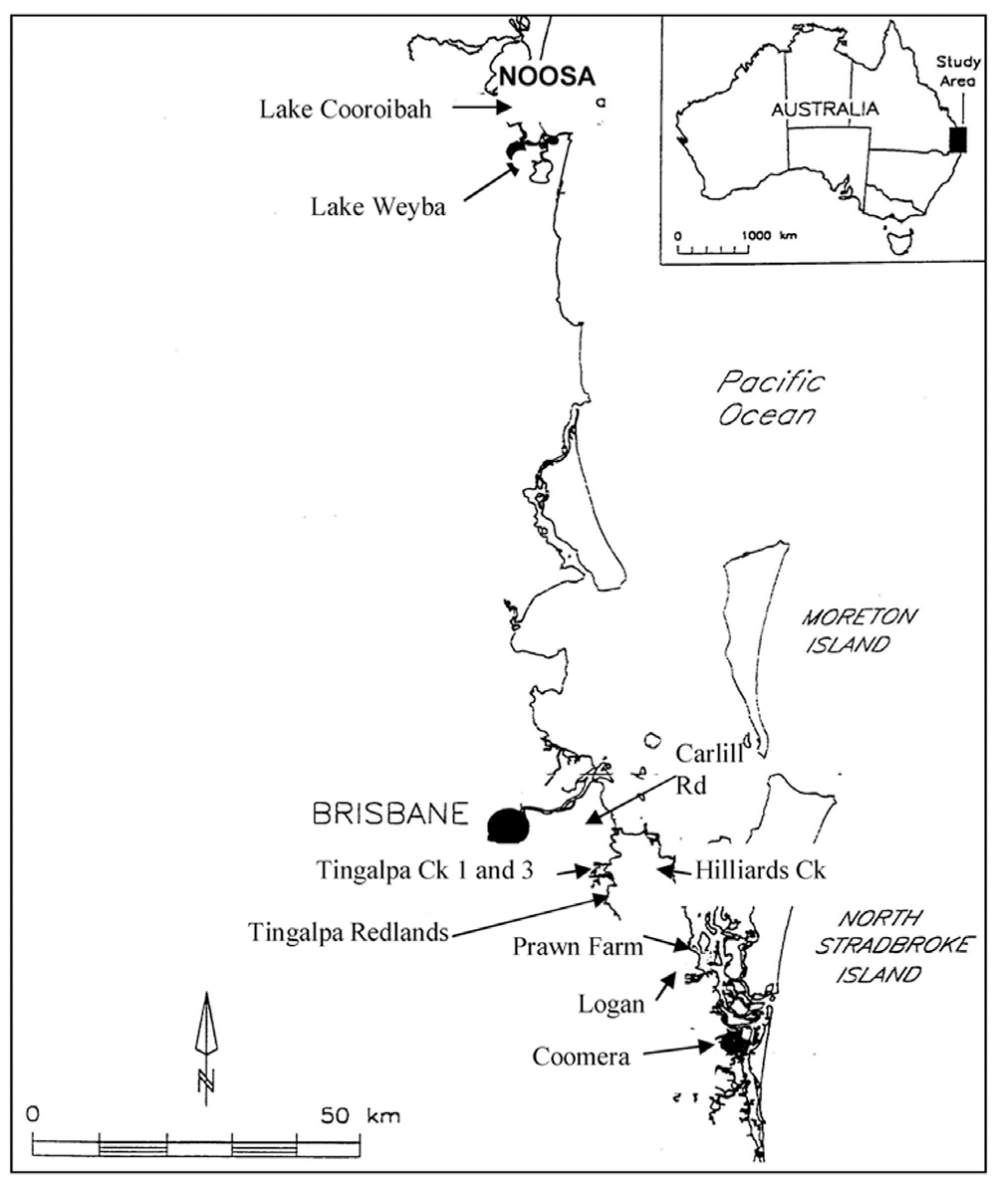
Location of study sites.

There are relationships between land cover (vegetation types or its absence) and oviposition (Dale et al. 1986a; Kay and Jorgensen 1986; Ritchie 1994; Ritchie and Jennings 1994). However most studies have focussed on individual sites and not the more general situation as reflected in a diversity of sites. The research aims are to assess the differences and similarities between a range of sites and to specifically explore relationships between eggshell distribution and both location on a marsh and land cover types. The research reported here includes sites ranging over a latitudinal distance of around 200 km.

## Materials and Methods

### Study areas

Ten sites were selected that contained undisturbed salt marsh and had previously been identified as productive mosquito habitats. They were also selected because they were candidates for habitat modification for mosquito control, and their oviposition characteristics were of interest to the local mosquito control agencies. They are in areas experiencing rapid population growth, are major tourist destinations, have considerable salt marsh mosquito populations and experience human cases of Ross River virus each year. These areas range from Noosa (26° 24′ S, 153° 04′ E) some 140 km north of the state capital, Brisbane, to just north of the Gold Coast some 80 km to the south at Coomera Island (27° 51′S, 153° 23′E). Approximate locations of the sites are shown on Figure 1.

### Data collection

Eggshell sampling and laboratory analysis followed the method of Ritchie and Jennings (1994) and Dale et al. (1999). Relative age of eggshells was recorded using a colour key provided by Ritchie (personal communication) but this information was only used to demonstrate that eggshells had accumulated over some length of time. Samples were collected between 1994 and 1999, mostly during the cooler months (June-September), when the marshes are relatively dry. Months and years are shown in Table 1 for each site. In brief, 15 × 15 cc random substrate sub-samples were pooled to make each sample (n = 40). Two replicates were collected at each sampling point. In all there were 80 samples. The sampling was stratified, where possible, by three locations within each marsh as shown in Table 1; low marsh, pool margins and the upper marsh surface between pools. For three sites not all three locations were sampled: there was no low marsh sample at Coomera and no pool edge at both Lake Cooroibah and Logan as the latter did not have clearly defined pools. Samples were assigned to a salt marsh land cover class based on the vegetation classes identified in Dale et al. (1986b) and extended to include the range of land covers across all ten sites. This resulted in 6 discrete classes identified by their dominant or co-dominant species (in terms of cover):

1. the grass, *Sporobolus virginicus*, (L.) Kunth (Poales: Poaceae)
2. beaded grasswort, *Sarcocornia quinqueflora*, (Bunge ex (Ung.-Stern) A.J. Scott (Caryophyllales: Chenopodiaceae)
3. *S. virginicus* mixed with *S. quinqueflora*
4. arrowgrass, *Triglochin striata* Ruiz and Pavn (Alismatales: Juncaginaceae), mixed with *S. virginicus*
5. Australian pine, *Casuarina* spp.
6. bare mud

**Table 1.**
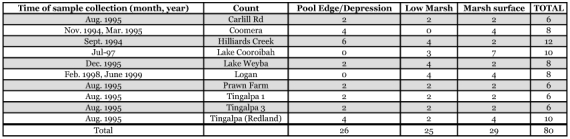
Number of sample sites by location and month/year of sample collection.

### Data analysis

Data were log transformed In(X+1) to approximate a normal distribution and analysed using ANOVA. Where there were significant differences identified in the ANOVA, extended t-tests (Student's t-) were used to identify the specific differences. To relate to the raw data the means of the transformed data analysis were converted back to the original scale via exp (mean) - 1 (exp is exponential). Where eggshell distribution was strongly related to land cover the relationship between the site and land cover was also assessed using *χ* ^2^ analysis.

**Table 2.**
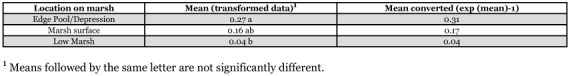
Eggshell distribution by location on salt marsh, for 10 south east Queensland salt marshes.

The data were also partitioned, with the transformed eggshell data as the dependent variable and land cover and location on the marsh as independent variables. The resulting tree structure provides a method to characterise sites in terms of their eggshell densities. The process starts with one group containing the dependent variable values (transformed eggshell counts). The program considers each independent variable (cover or location). It calculates the value (in this case it is a nominal value as the data are characters) for the independent variable/s that optimally splits the whole group into two groups. This splitting continues with each step, choosing the best split each time.

All analyses were conducted using JMP (The Statistical Discovery Software) package (version 5.0.1.2) (SAS Inst. Inc.).

## Results

There was evidence that eggshells had remained in the sites over a period of time. The distribution over the 4 age classes from oldest to youngest was 12.3%, 31.7%, 39.2% and 16.8% in the youngest class. There was no significant relationship between eggshell relative age and either location on the marsh or land cover. There were significant differences in eggshell distribution between the sites (F= 4.59, df 9, 70, P< 0.001). The extended t-test showed that the Hilliards Creek differed significantly from all the others. Repeating the analysis excluding the Hilliards Creek site resulted in significant differences among the remaining sites (F = 2.87 df 8, 59, P<0.01). The extended t-test results showed that the Prawn Farm, Lake Cooroibah and Lake Weyba sites had significantly more eggshells than the others (t = 2.00, P<0.05). There appeared to be no spatial pattern to the eggshell distribution between sites.

**Table 3.**
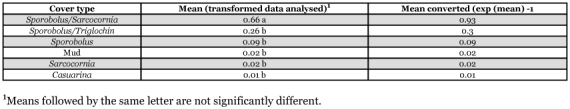
Relative abundance of *Aedes vigilax* eggshells by cover type, for 10 south east Queensland salt marshes.

Eggshell distribution was significantly related to location on the marsh as shown in Table 2 (F = 3.81, df 2, 77, P<0.05). Pool edges and depressions had significantly higher numbers of eggshells followed by marsh surface and both of those had significantly higher numbers than the low marsh.

**Figure 2.**
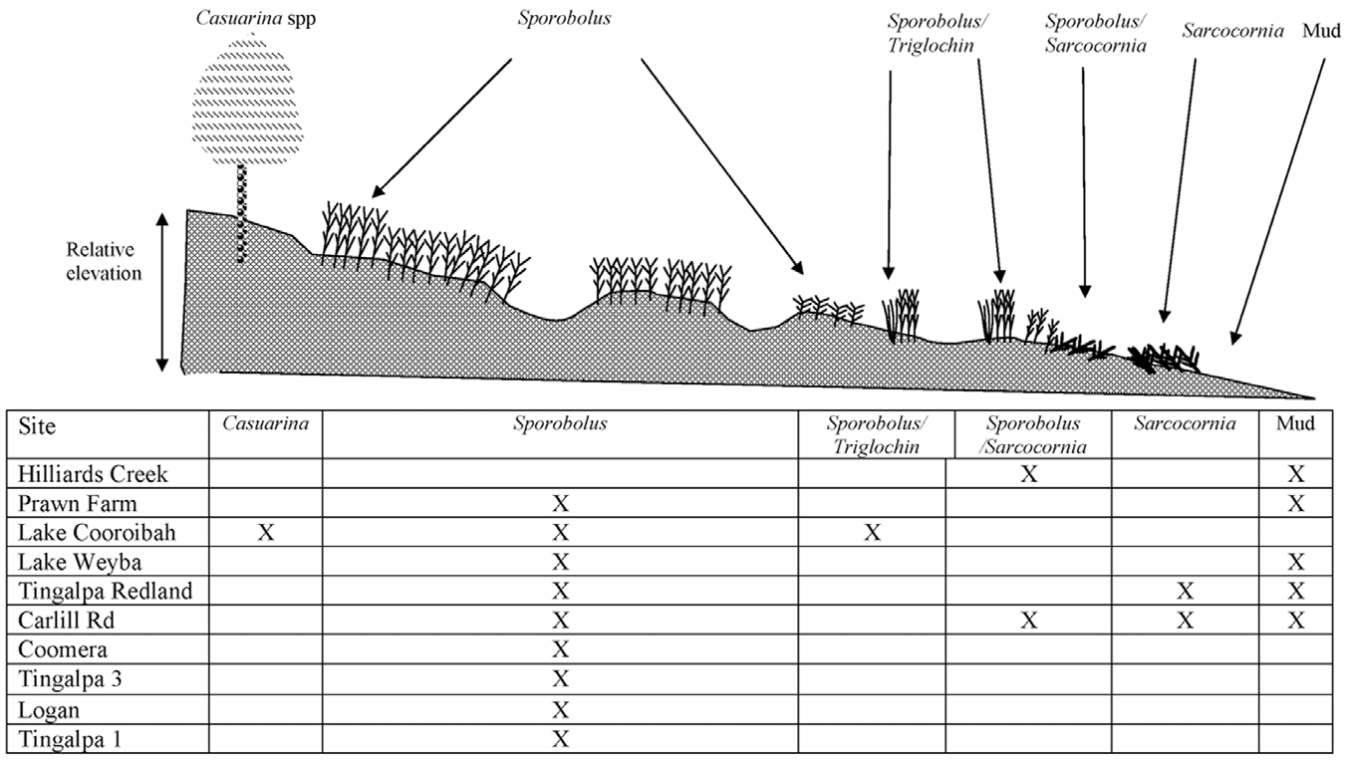
Schematic diagram of site characteristics and dates of sample collections. Sites are ranked from highest eggshell densities (Hilliards Creek) to lowest (Logan).

There was also a significant relationship between land cover and eggshell distribution as shown in Table 3 (F = 12.63, df 5, 74, P<0. 001). Figure 2 shows the distribution of land cover types by site. In brief, the *Sporobolus/Sarcocornia* mix contained significantly more eggshells than other types, followed by a mix of *Sporobolus/Triglochin*. Those two vegetation mixes were present at two of the most productive sites: Hilliards Creek and Lake Cooroibah (Figure 2). The *Sporobolus/Sarcocornia* mix was also present at the Carlill Rd site but, at that site, there was no relationship between land cover and eggshell numbers (P = 0.68). The other sites with large numbers of eggshells were dominated by *Sporobolus*, but this alone does not account for the differences between sites. This then may be explained by location on the marsh as described below. In contrast, *Ae. vigilax* eggshells were absent in 75% of the *Casuarina* sp. samples. Mud samples occasionally contained eggshells (13 out of the total of 80), but mud was often in the low marsh and this area was generally not productive.

*χ*^2^ analysis indicated a significant relationship between location on the marsh and land cover (*χ* ^2^ = 56.52, df 10, 65, P<0.0001). The low marsh was dominated by mud (52% of sites) and *Sporobolus* and *Sporobolus/Triglochin* (together 48% of sites). Pool edge sites were predominantly *Sporobolus* (58% of sites) with smaller proportions of *Sporobolus/Sarcocornia* (29%) and of *Sporobolus/Triglochin* (13%). Marsh surface sites were mainly covered in *Sporobolus* (65%) with smaller proportions of sites with *Sporobolus/Sarcocornia* (13%), *Casuarina* (13%), *Sarcocornia* (6%) and *Sporobolus/Triglochin* (3%).

**Figure 3.**
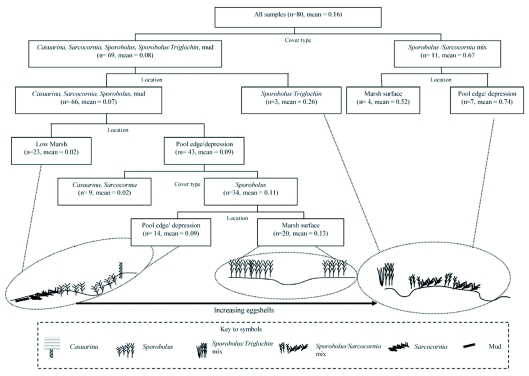
Partition analysis and habitat characteristics (with number of samples and means (transformed data).

The partition analysis identified both land cover and location on the marsh as important indicators of eggshell distribution. The analysis provided a tree that summarised the findings and identified the characteristics that best related to places that would be likely to have (or not have) large numbers of eggshells (Figure 3). At the highest level cover type was most important, with the first split distinguishing *Sporobolus/Sarcocornia* from all other covers. The right hand side is associated with large eggshell numbers. The *Sporobolus/Sarcocornia* mix is most important and, within that cover type, there is a further split, such that location on pool edge or depression was likely to be more productive of eggshells than the marsh surface. The left side of the tree generally identifies characteristics likely to yield fewer eggshells, splitting on cover and separating the effects of different types. However, although on the left side, the *Sporobolus/Triglochin* cover splits at a high level from other types with somewhat higher eggshell counts. As well, on the left side there is a lower level split on location on the marsh with pool edge and marsh surface locations yielding eggshells, but *only* if also associated with *Sporobolus*, and, within the *Sporobolus*, mainly if also associated with a marsh surface location.

## Discussion

It was clear that the sites were producers of larvae over at least several years, as evidenced by the relative ages of the eggshells. This is useful information for management as it helps focus control on areas that are consistent problems. There were large differences between the sites in terms of numbers of eggshell. However, overall, although location on the marsh was important, it was land cover that was the most significant factor for eggshell distribution, as indicated by the high level split in the partition analysis (Figure 3). They were most numerous in *Sporobolus* mixed with either *Sarcocornia* or, but less significantly, with *Triglochin*. Both *Sarcocornia* and *Triglochin* tend to occupy wetter sites, closer to the tidal source and this is important for the ultimate survival of larvae (Dale et al. 1986a). It also appears that numbers of eggshells related to land cover may differ between the sites. The relatively high yield of eggshells at the Prawn Farm and Lake Weyba sites was not related to the two land cover classes most significantly associated overall with eggshells, but to *Sporobolus*, with the majority of *Sporobolus* samples located on the marsh surface or at the edge of pools/depressions. All sites contained *Sporobolus*. Thus, that species alone does not appear to distinguish between sites in terms of eggshell distribution, perhaps because it is found at all locations in the study sites. This may be because it is morphologically plastic and responds to changes in environmental conditions, mainly salinity, by altering its leaf size (Blits and Gallagher 1991) and its overall density. These factors may in turn affect its attractiveness for a gravid mosquito. For example, the substrate may be more accessible under the short-leaved and sparse form and this may explain why the mix of *Sporobolus* and *Sarcocornia* is important for eggshells. *Sarcocornia* tends to occupy lower, more saline, locations that are frequently flooded. Overall the results agree with other Australian research that found associations between oviposition and vegetation in sub tropical Southeast Queensland (Dale et al. 1986a; Kay and Jorgensen 1986). For example several reports have found positive associations between eggshells and percent cover of *Sporobolus* and *Sarcocornia* in a more temperate situation in New South Wales (e.g.,Gislason and Russell 1997; Turner and Streever 1997b). A pool edge location is also important as it satisfies the sequence of wet-dry-wet conditions necessary for egg conditioning, hatching and larval survival, as larvae are flushed into pools on a flooding tide (Dale et al. 1986a).

Low marsh close to the tidal source had the least number of eggshells and this is generally consistent with the observations of others for *Ae. vigilax* (Ritchie and Jennings 1994; Dale et al. 1986a; Turner and Streever 1997b; Dale et al. 2002). However if marsh wetness increases, for example related to sea level, climate change or other factors, then the low marsh environment may encroach inland and the oviposition characteristics may change, though the nature of change would depend on particular marsh topography and cover. There is already evidence that mangroves, that are low on the intertidal profile, are encroaching on salt marsh in eastern Australia (McTainsh et al. 1986; Saintilan and Williams 2000; Jones et al. 2004).

The partition analysis simplified and summarised the findings and provided a map to quickly locate likely high producers of eggshells, at least in Southeast Queensland. So the simple route to locate high eggshell density would be to first identify cover type as a mix of *Sporobolus* and *Sarcocornia* and, secondly if present, look for an association with pool edge/depression or marsh surface. A subsidiary query would be to look at pool edge and depression location and ask if it contains *Sporobolus*, as that location and vegetation combination did have eggshells, though not in very large numbers.

New information from this research supports the concept that marshes are highly heterogeneous (Dale and Hulsman 1988) and the differences in eggshell distribution and land cover between different areas illustrate this. The characteristics that are likely to have large numbers of eggshells include many pools/depressions, and vegetation mixes of *Sporobolus* with *Sarcocornia* or *Triglochin*. Conversely, larger areas of low marsh and relatively flat marsh surfaces had fewer eggshells. An emerging issue is one of marsh and subsequent mosquito response to environmental changes that may affect the factors related to oviposition such as low marsh extent and land cover changes. Our data for example should aid in extrapolating likely scenarios for *Ae. vigilax* oviposition in response to sea level rises and climate change, and in planning future land use and its public health implications.
